# Removal of Nickel Ions from Aqueous Solutions by 2-Hydroxyethyl Acrylate/Itaconic Acid Hydrogels Optimized with Response Surface Methodology

**DOI:** 10.3390/gels7040225

**Published:** 2021-11-22

**Authors:** Katarina Antić, Antonije Onjia, Dana Vasiljević-Radović, Zlate Veličković, Simonida Lj. Tomić

**Affiliations:** 1 Innovation Center of Faculty of Technology and Metallurgy, University of Belgrade, Karnegijeva 4, 11000 Belgrade, Serbia; 2 Faculty of Technology and Metallurgy, University of Belgrade, Karnegijeva 4, 11000 Belgrade, Serbia; onjia@tmf.bg.ac.rs (A.O.); simonida@tmf.bg.ac.rs (S.Lj.T.); 3 Institute of Chemistry, Technology and Metallurgy, University of Belgrade, Njegoseva 12, 11000 Belgrade, Serbia; dana@nanosys.ihtm.bg.ac.rs; 4 Military Academy, University of Defense, Veljka Lukica Kurjaka 33, 11000 Belgrade, Serbia; zlate.velickovic@va.mod.gov.rs

**Keywords:** hydrogel, nickel removal, response surface methodology, itaconic acid, desorption

## Abstract

The adsorption of Ni^2+^ ions from water solutions by using hydrogels based on 2-hydroxyethyl acrylate (HEA) and itaconic acid (IA) was studied. Hydrogel synthesis was optimized with response surface methodology (RSM). The hydrogel with the best adsorption capacity towards Ni^2+^ ions was chosen for further experiments. The hydrogel was characterized by Fourier transform infrared spectroscopy (FTIR), scanning electron microscopy (SEM) and atomic force microscopy (AFM) analysis before and after the adsorption of Ni^2+^ ions. Batch equilibrium experiments were conducted to investigate the influence of solution pH, hydrogel weight, ionic strength, adsorption time, temperature and initial concentration of nickel ions on the adsorption. Time-dependent adsorption fitted the best to the pseudo-second-order kinetic model. A thermodynamic study revealed that the adsorption was an exothermic and non-spontaneous process. Five isotherm models were studied, and the best fit was obtained with the Redlich–Peterson model. Consecutive adsorption/desorption studies indicated that the HEA/IA hydrogel can be efficiently used as a sorbent for the removal of Ni^2+^ ions from the water solution. This study develops a potential adsorbent for the effective removal of trace nickel ions.

## 1. Introduction

Wastes produced in different kinds of industries have become an important environmental problem at the global level [1,2]. Nickel is widely used in heavy metals that has many beneficial applications in human life, but it is also very dangerous if dispatched into natural water [3,4]. More than half of nickel production all over the world is used in stainless steel production. Nickel is also used in the production of metallic alloys, nonferrous metals, mineral processing, paint formulation, electroplating, batteries manufacturing, forging and production of coins [5,6,7].

Due to its very high toxicity, wastewater generated from nickel processing should be appropriately treated before final discharge into the environment. There are several techniques for removing nickel ions from water: chemical precipitation, ion exchange, adsorption, membrane filtration and reverse osmosis [8]. In recent years, the adsorption process has been widely used for the removal of heavy metals from water due to its high efficiency, ease of operation, cost-effectiveness and no secondary pollution [9,10,11,12]. Hydrogels as frequently used adsorbents also attract interest due to properties such as tunable design, easy handling and high adsorption capacity [2]. Hydrogels containing different functional groups such as carboxylic (–COOH) or amino (–NH_2_) groups are becoming more interesting for applications in water treatment.

Response surface methodology (RSM) is a collection of statistical and mathematical techniques useful for modeling and for the analysis of problems in which a response of interest is influenced by several variables [13,14]. RSM reduces the number of experiments and delivers an appropriate model [15,16,17]. Therefore, RSM has been used successfully in chemical engineering and environmental fields [16,17,18]. In the literature, RSMs based on Central Composite Design (CCD), D-Optimal design and Box–Behnken design have been used in various engineering applications, including optimization of heavy metal removal conditions, dye, pesticides from wastewater and drinking water, electrocoagulation, optimization of synthesis conditions of different adsorbents, optimization of conditions for encapsulation of probiotics, etc. [13,19,20,21,22,23]. RSM has also been successfully and widely applied in water and wastewater treatment optimization [24]. RSM has been effectively applied in the optimization of treatment processes such as industrial paint wastewater, textile dye wastewater, tannery wastewater, landfill leachate, olive oil effluent and palm mill effluent [25,26,27,28,29,30,31]. Using RSM in water and wastewater treatment process optimizations could contribute to significant improvements in removal efficiency and operational cost reduction.

The objective of our study was to synthesise a hydrogel based on 2-hydroxyethyl acrylate and itaconic acid with good removal ability for Ni^2+^ ions. The optimization of HEA/IA hydrogel synthesis was performed by using response surface methodology (RSM). In order to explore the adsorption ability and mechanism of HEA/IA hydrogels, the characteristics of Ni^2+^ adsorption process on the hydrogels were investigated. The adsorption abilities and mechanisms of heavy metal adsorption on the synthesized hydrogels were investigated by using FTIR, SEM and AFM, before and after Ni^2+^ sorption. The effects of initial pH of the water solution, adsorbent mass, temperature and influence of salts on the nickel ions’ adsorption on hydrogels were also investigated. Additionally, the adsorption kinetics and isotherms of Ni^2+^ ions on HEA/IA were analyzed in addition to the desorption and reusability of hydrogels.

## 2. Results and Discussion

### 2.1. Optimization of Hydrogel Synthesis

**Figure 1:**
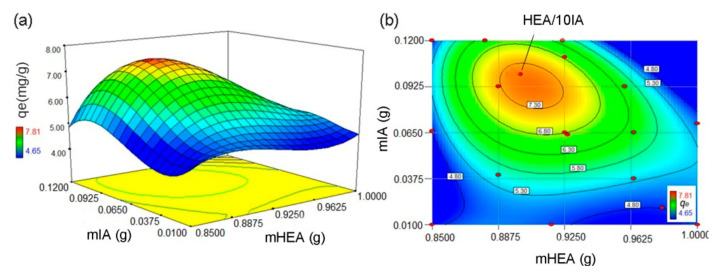
The estimated surface (**a**) and contour (**b**) plots based on *q_e_* (mg/g) for Ni^2+^ ions.

The optimization of hydrogel synthesis was performed in order to produce adsorbents with good swelling properties and removal abilities for Ni^2+^ ions. The results of the optimization of adsorbent synthesis, conducted based on the experimental plan, are shown in Supplementary Materials (Table S1). The estimated surface Figure 1a and contour Figure 1b plots based on *q_e_* (mg/g) for Ni^2+^ ions are presented in Figure 1. The *x* and *y* axes show the two input parameters for hydrogel synthesis, which have the greatest influence on the adsorption properties of the hydrogel, mass of 2-hydroxyethyl acrylate (HEA) and itaconic acid (IA). The third axis represents the response–adsorption capacity of the hydrogel towards Ni^2+^ ions, which depends on the composition of the hydrogel itself. As presented in Figure 1, Figures S1−S5 and Table S1, the maximum adsorption capacity for Ni^2+^ ions was achieved for hydrogel prepared by using 10 mass % of IA. In this paper, this sample was coded as HEA/10IA hydrogel. According to this result, all further experiments were performed with HEA/10IA hydrogel. Additionally, the interactions between process variables and responses were determined by analysis of variance (ANOVA), and the results are provided in Supplementary Materials (Table S2).

### 2.2. The Influence of pH, Adsorbent Weight and Ionic Strength on Adsorption of Ni^2+^ Ions

The pH of the solution is a fundamental parameter in the adsorption process of heavy metal ions.

**Figure 2:**
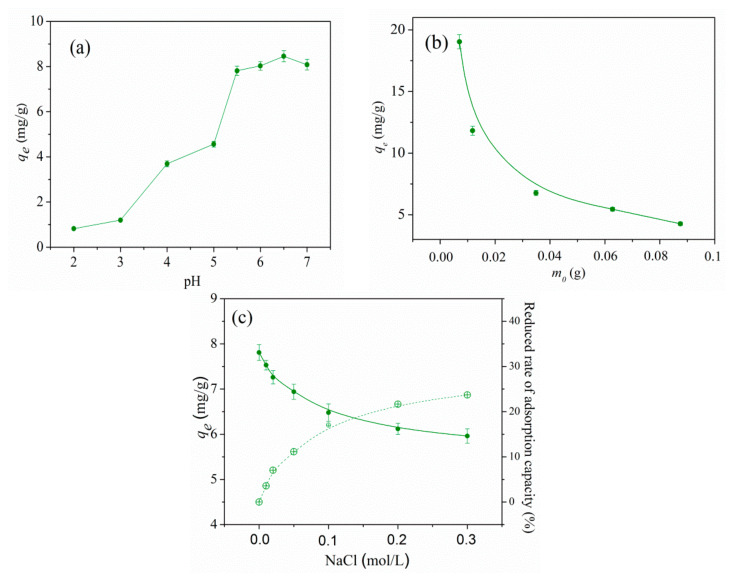
The influence of pH (**a**), adsorbent weight (**b**) and ionic strength (**c**) on the adsorption capacity of Ni^2+^ ions on HEA/10IA hydrogel.

Figure 2a presents how different values of pH of the solution affect the Ni^2+^ ion adsorption. The influence of pH value of the solution was investigated on different values, from 2.0 to 7.0 [4]. It can be noted that in acidic environments (pH < 3), the adsorption capacity is lower. This can be explained by the low dissociation of carboxylic groups in itaconic acid that results in competition between H^+^ and Ni^2+^ ions for the same sorption site. The adsorption of Ni^2+^ ions increases significantly with the increase in pH value. As expected, at pH values higher than 5.5, the adsorption of nickel ions increases due to the ionization of both carboxylic groups of itaconic acid. The negative charge density on the adsorbent increases, and electrostatic attraction between positively charged nickel ions and negatively charged binding sites result in an increase in adsorption. All experiments were conducted at pH 5.5 in order to avoid nickel ion precipitation.

Figure 2b represents the influence of adsorbent mass on the adsorption capacity of nickel ions. When adsorbent mass increased, adsorption capacity significantly decreased, which can be explained by the fact that nickel ions on the surface of the adsorbent and nickel ions in the solution reached a balance [32]. The influence of ionic strength on adsorption was also studied since different salts are very often found in water. The experiments were conducted with different concentrations of NaCl. It was found that the increase in ionic strength results in a decrease in the adsorption capacity of hydrogels (Figure 2c). It can be concluded that the increase in salt concentration in the solution blinds the electrostatic interactions of the carboxylic groups of itaconic acid, which results in a reduction in polymer chains [33,34].

### 2.3. Characterization of Ni^2+^ Loaded HEA/10IA Hydrogel

**Figure 3:**
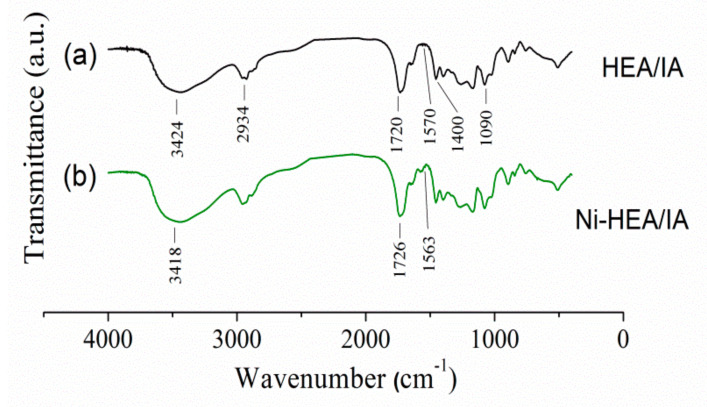
(**a**) FTIR spectra of the HEA/10IA hydrogel before (**a**) and after (**b**) Ni^2+^ ion adsorption.

The FTIR spectra of HEA/10IA hydrogel, before and after nickel adsorption, are presented in Figure 3. The wide peak at 3424 cm^−1^ represents stretching vibrations of –OH bond, and the peak intensities at 1400 and 1180 cm^−1^ demonstrate this too. Peaks at 2871 and 2934 cm^−1^ represent C–H stretch vibrations. Carboxylic groups exhibit a characteristic peak at 1720 cm^−1^, which is assigned to C=O stretching vibrations.

After the adsorption of nickel ions, the absorption band’s intensity was moved to higher or lower wavenumbers. The wide peak at 3424 cm^−1^ that represents stretching vibrations of –OH bond shifted to 3418 cm^−1^. An intensive peak at 1720 cm^−1^ was moved to a higher wavenumber (1726 cm^−1^), which could be explained by the interaction between COO^−^ and nickel ions. After nickel adsorption, the absorption band at 1570 cm^−1^, which is explained by the asymmetric stretching vibrations of C=O, moved to 1563 cm^−1^. All of these changes suggest that –OH and –COOH groups in the HEA/10IA hydrogel participated in the adsorption of nickel ions onto hydrogel. Based on all these facts, it can be concluded that –OH and –COOH groups are involved in Ni^2+^ ions adsorption [11].

**Figure 4:**
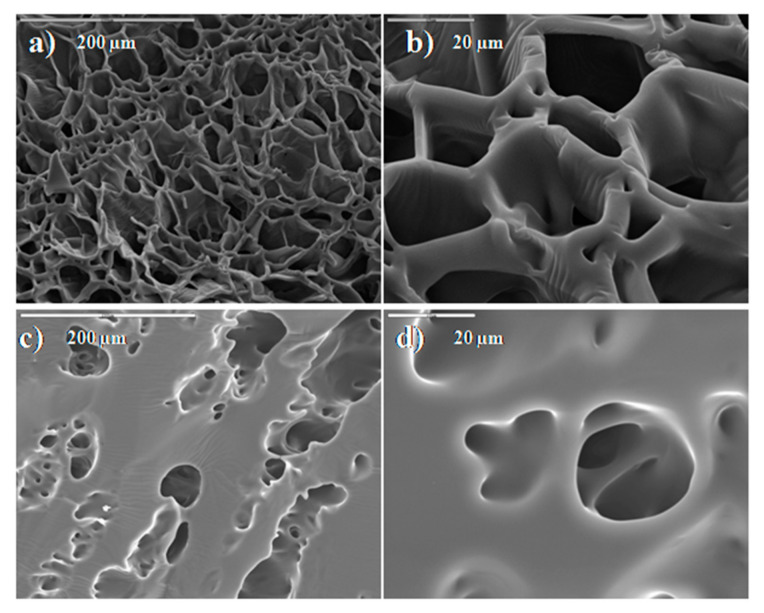
SEM images: morphology of a cross section of (**a**) HEA/10IA (bar 200 μm) and (**b**) HEA/10IA (bar 20 μm); hydrogel swollen in deionized water; (**c**) SEM micrographs of HEA/10IA hydrogel with adsorbed Ni^2+^ ions (bar 200 μm); (**d**) (bar 20 μm).

The SEM images of HEA/10IA hydrogel, before and after adsorption are presented in Figure 4. The structure of hydrogel before adsorption in Figure 4a,b is very porous and has a typical honeycomb structure. After nickel ions adsorption, the pores of the hydrogel become smaller, and their structure becomes rougher (Figure 4c,d). During adsorption processes, nickel ions interact with carboxyl groups, and electrostatic repulsive forces between the COO^−^ groups tend to decrease, which results in a reduction in hydrogel pores.

**Figure 5:**
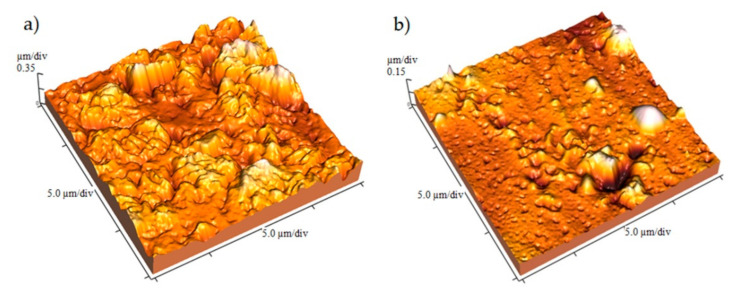
AFM images of the top surface of HEA/10IA hydrogel: (**a**) before adsorption; (**b**) after Ni^2+^ adsorption.

In order to observe surface texture and roughness characteristics, AFM images of 3D surface topography of hydrogel before and after nickel adsorption were registered (Figure 5). The micrograph of the nickel-free sample represents a predominantly hill-valley-structured surface with various size grains that are randomly aggregated (Figure 5a). The change in surface topography can be observed after nickel adsorption. The surface of hydrogel is significantly smoother, which could be explained by the fact that the surface has adsorbed metal ions.

### 2.4. Adsorption Isotherms

**Figure 6:**
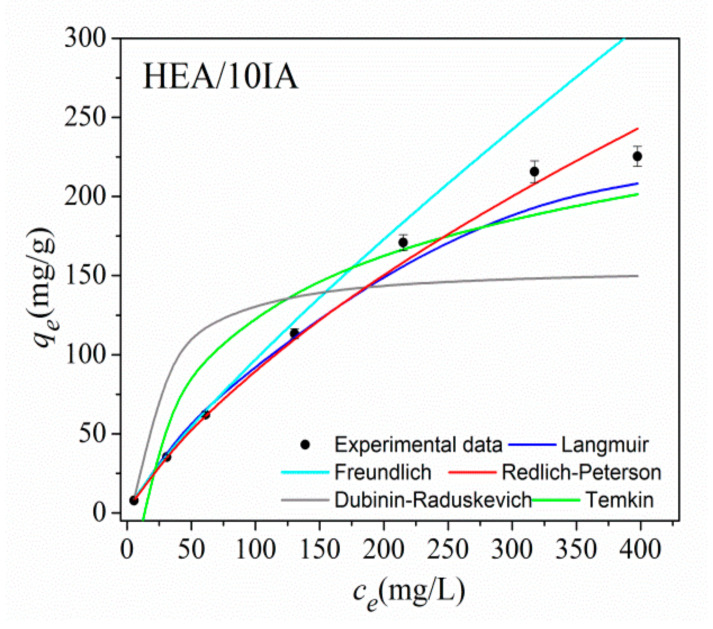
Comparison of five isotherms employed in this work for the Ni^2+^ adsorption onto HEA/10IA hydrogel.

The adsorption isotherms of Ni^2+^ onto HEA/10IA hydrogel, as the dependence of *q_e_* on the equilibrium nickel concentration *c_e_*, are presented in Figure 6. All experimental data were fitted with five isotherms (Langmuir, Freundlich, Redlich–Peterson, Temkin and Dubinin–Radushkevich) by using commercial software OriginPro 8.5. The applicability of the isotherm models for describing the adsorption process was judged by a correlation coefficient, (*R*^2^) and chi-square analysis (*χ*^2^). The obtained characteristic parameters of the evaluated isotherm models are summarized in Table 1.

The Langmuir, Freundlich and Redlich–Peterson isotherms demonstrated high *R*^2^ values; thus, it was difficult to conclude which model provides the best interpretation of experimental data. With this in mind, a chi-square test was used to determine the best isotherm model for the interpretation of experimental data. The advantage of using the chi-square test was in comparing all isotherms on the same abscissa and ordinate.

The chi-square test measures the difference between experimental and model data. The mathematical form of a chi-square is given as follows [35]:

(1) $$\chi^{2}=\sum \frac{{\left(q_{e,exp}-q_{e,cal}\right)}^{2}}{q_{e,cal}}$$

where *q_e,exp_* is experimental equilibrium capacity data, and *q_e,cal_* is the equilibrium capacity from the isotherm model. If data from the model are similar to experimental data, *χ*^2^ will be small and vice versa.

Based on the *χ*^2^ values, the best interpretation of the experimental data was provided by the Redlich–Peterson isotherm. Dubinin–Radushkevich and Tempkin models had a much lower agreement with experimental data than compared to Langmuir, Freundlich and Redlich–Peterson models. The Langmuir equation can be expressed in terms of dimensionless separation factor, *R_L_*, which indicates whether the sorption was favorable (0 < *R_L_* < 1), unfavorable (*R_L_* > 1), linear (*R_L_* = 1) or irreversible (*R_L_* = 0), and it is provided by Equation (2):

(2) $$R_{L}=\frac{1}{1+K_{L}C_{o}}$$

where *C*_o_ is the maximal initial metal ion concentration (mg/L) [36].

The values of the separation factors are presented in Table 2. The values of the separation factors 0 < *R_L_* < 1 and the value for the Freundlich exponent *n* > 1 indicated that the sorption of Ni^2+^ onto investigated hydrogels was favorable. The calculated *R_L_* values for all studied models point out that sorption is more favorable at higher initial metal ion concentrations.

The values of the adsorption capacity obtained by D–R equation are higher than the *q_m_* values obtained by the Langmuir model, which is expected because the D–R model takes into account the porous structure of the adsorbent. Adsorption capacities for Ni^2+^ ions on different sorbents reported in the literature are presented in Table 3.

### 2.5. Adsorption Kinetics and Thermodynamics Studies

The experimental results were fitted with two main types of kinetic models: the pseudo-first-order model and pseudo-second-order model [42,43]. The linear forms of pseudo-first-order and pseudo-second-order models are presented in Equations (3) and (4), respectively:

(3) $$log\left(q_{e}-q_{t}\right)=logq_{e}-\frac{k_{1}}{2.303}t$$

(4) $$\frac{t}{q_{t}}=\frac{1}{k_{2}q_{e}^{2}}+\frac{1}{q_{e}}t$$

where *k*_1_ (h^−1^) and *k*_2_ (g mg^−1^ h^−1^) are the pseudo-first and the pseudo-second-order rate constants, and *q_e_* and *q_t_* (mg/g) are the adsorption capacities at equilibrium and at time *t* (h), respectively.

The predicted *q_e_* values as rate constants *k*_1_ and *k*_2_ were determined from the slope and intercept of plots in (*q_e_* − *q_t_*) vs. *t* and *t/q_t_* vs. *t*, respectively. The results for the pseudo-first-order and pseudo-second-order models are presented in Table 4.

The adsorption of nickel ions was well adjusted to the pseudo-second-order kinetic model, according to the correlation coefficients (*R*^2^ > 0.99), and the comparison between the calculated equilibrium adsorption capacity (*q_e,cal_*) and the experimental adsorption capacity (*q_e,exp_*). Accordingly, the adsorption of nickel ions on HEA/IA might be a chemical process induced by sharing electrons between the HEA/IA and the nickel ions [37,44].

In order to examine the influence of temperature on the adsorption process, adsorption experiments were performed at three temperatures 10, 25 and 50 °C at a pH value of 5.5, fixed nickel ion concentration and adsorbent dosage of 0.030 g.

The basic thermodynamic parameters are calculated from the thermodynamic equilibrium constant, *K_c_*. The standard Gibbs free energy ∆*G*^o^ (kJ/mol), standard enthalpy change ∆*H*^o^ (kJ/mol) and standard entropy change ∆*S*^o^ (J/mol K) were calculated by using the following equations:

(5) $$K_{c}=\frac{C_{s}}{C_{e}}$$

(6) $$\Delta G^{o}=-RTlnK_{c}$$

(7) $$lnK_{c}=\frac{\Delta S^{o}}{R}-\frac{\Delta H^{o}}{RT}$$

where *K_c_* is the equilibrium constant, *C_s_* is the amount of metal ions sorbed at equilibrium (mg/L), *C_e_* is the amount of metal ions remained in the solution (mg/L), *R* is the universal gas constant (kJ/mol K) and *T* is the solution temperature (K) [45].

∆*S*^o^ and ∆*H*^o^ values have been delivered from the slope and intercept of a plot ln *K_c_* vs. *1/T* based on Equation (7). ∆*G*^o^ values were determined from the two parameters by using Equation (6).

With the increase in temperature, Ni^2+^ ions adsorption decreases (Table 5), implying that the adsorption of nickel onto HEA/10IA hydrogels is an exothermic process. This could also be supported with the fact that the value of *∆H*^o^ is negative. Based on the values of ∆*G*^o^ that are positive, it can be concluded that the adsorption is non-spontaneous, and the degree of the spontaneity of the reaction decreases with temperature increases. The ∆*S*^o^ value is negative, suggesting that there is a decrease in randomness at the solid–solution interface of Ni^2+^ ions onto the HEA/10IA hydrogel [46].

### 2.6. Desorption and Regeneration Studies

Desorption of Ni^2+^ ions was performed with 0.1 mol/L solution of HNO_3,_ HCl and CH_3_COOH. The best desorption results were obtained with HNO_3_ at 88.1%, 62.3% with HCL and only 57.2% with CH_3_COOH.

**Figure 7:**
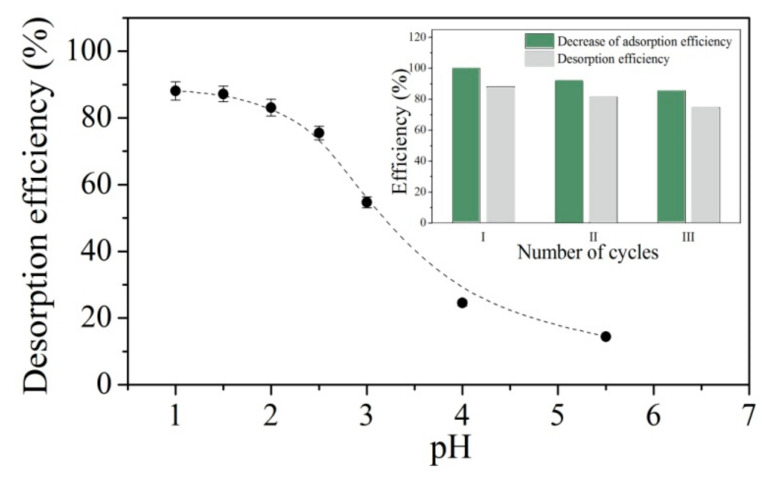
Desorption of Ni^2+^ ions from HEA/10IA hydrogel.

To examine the influence of pH of the desorption solution, experiments were conducted with different concentrations of HNO_3_, and the results are presented in Figure 7. The efficiency of desorption decreased with the increase in pH of the desorbing solution.

In order to evaluate the potential reusability of hydrogels, three successive sorption–desorption cycles were repeated. It was found that by the end of the third cycle, 85.2% of the initial adsorption capacity was obtained.

## 3. Conclusions

2-Hydroxyethyl acrylate-itaconic acid hydrogels were synthesized, and RSM was used for the optimization of adsorbent synthesis. The ability of optimized hydrogel for the removal of Ni^2+^ ions from the water was investigated. By using RSM, we have reduced the number of experiments and found the best combination of monomers for removing nickel ions. It was found that the solution’s pH, initial concentration of metal ions, the adsorbent mass and ionic influenced the adsorption process. By investigating FTIR spectra, it can be concluded that –OH and –COOH groups are involved in the adsorption process. Moreover, the adsorption of nickel ions is favorable and can be fitted by the pseudo-second-order rate equation and Redlich–Peterson isotherm model, respectively. By investigating the desorption process, it could be concluded that the desorption of HEA/IA hydrogel is pH-dependent, and hydrogels could be efficiently desorbed with 0.1 mol/L HNO_3_. After the three adsorption–desorption cycles performed, it could be concluded that the hydrogels possess high potentials for desorption and reuse.

## 4. Materials and Methods

### 4.1. Materials

The monomers, 2-Hydroxyethyl acrylate (HEA) and Itaconic acid (IA) were supplied from Sigma-Aldrich, Germany and Fluka, Germany, respectively. Cross-linker-Ethyleneglycol dimethacrylate (EGDMA), initiator-potassium persulfate (KPS) and activator -*N*,*N*,*N*′,*N*′-tetramethylethylene diamine (TEMED) were obtained from Sigma-Aldrich, Germany. All chemicals were used as received. The solution of Nickel (1000 mg/L) was prepared with Ni(NO_3_)_2_·6H_2_O (Merck, Germany). All polymerizations and solution preparations were performed with distilled water.

### 4.2. Hydrogel Synthesis

The hydrogel was synthesized by using comonomers IA and HEA with 10 mass % of IA. The hydrogel was synthesized by radical copolymerization of HEA and IA and cross-linking with ethyleneglycol dimethacrylate (EGDMA). Potassium persulfate (KPS) was used as initiator, and *N*,*N*,*N*′,*N*′-tetramethyl ethylene diamine (TEMED) was used as an activator. The reactants were dissolved in a mixture of water/ethanol. Reactants were mixed and degassed for 15 min. After degassation, the mixture was poured out into glass moulds that were 2 mm wide. The moulds were placed in a dryer with a constant temperature of 50 °C for 24 h. After gelation, fresh gels were cut into disks. All discs were immersed in distilled water for 7 days in order to remove unreacted chemicals. The distilled water was changed every day. The disks were taken out of the water and dried to xerogels at room temperature. The xerogel disks used in all experiment were of average diameters of 0.450 ± 0.010 cm and 0.150 ± 0.010 cm with respect to averages in thickness.

### 4.3. Optimization of Hydrogel Synthesis

The optimization of the ratio of the mass of the reactants to the reaction mixture was performed in order to produce a highly efficient hydrogel that could be applied for the removal of heavy metal ions from water. The most influential operating parameters in the synthesis of hydrogel-based adsorbents and the ratio of HEA and IA were selected in the optimization process in order to achieve the goals of optimizing high adsorption capacities.

The optimization of the hydrogel synthesis was carried out by using surface response methodology (RSM), according to two factors D-optimal design. RSM is in accordance with the basic principles of environmental protection, where there is a significant reduction in the number of experiments, resulting in a decrease in waste production. An experimental plan for response surface methodology (RSM) applied for the optimization of hydrogel synthesis is provided in Supplementary Materials (Table S1).

All experiments, excluding the central point, were carried out in duplicate. In order to synthesise hydrogel with good removal efficiency, the adsorption capacity was chosen as the output variable. A second-order polynomial equation was used for fitting the experimental data. The coefficients of the response function and their statistical significance were determined by the least squares method by using commercial software Design-Expert, Software Version 9 (Stat-Ease, Inc. 2021 E. Hennepin Ave. Suite 480, Minneapolis, MA, USA).

### 4.4. Hydrogels Characterisation

Fourier transform infrared spectroscopy (FTIR) of unloaded and loaded hydrogel samples was recorded in transmission mode by using a Bomem MB 100 FTIR spectrophotometer, as KBr pellets. Before recordings, xerogels were crumbled into powder and mixed with potassium bromide (Merck IR spectroscopy grade) in the proportion of 1:100 and then compressed into a 12 mm semi-transparent disk under pressure (Pressure gage, Shimadzu). The morphology of the hydrogel samples before and after nickel adsorption was observed on a scanning electron microscope (SEM) instrument, JEOL JSM-5800. The surface topography changes before and after adsorption were observed and recorded by atomic force microscopy (AFM) in the contact mode bt using Auto Probe CP Research (TM Microscopes—Veeco Instruments, Santa Barbara, CA, USA). For an estimation of the roughness of the samples by the determination of the arithmetic average of the absolute (Ra) roughness parameters, the software SPMLab (SPMLab NT Ver. 6.0.2., Veeco Instruments, Santa Barbara, CA, USA) was used.

The ICP-OES Thermo iCAP 6500 system, equipped with the Thermo iTEVA software, a concentric nebulizer and a Cyclonic Spray Chamber, was used for the determination of the concentration of Ni^2+^ ions in the solution.

### 4.5. Adsorption Experiments

To evaluate the Ni^2+^ removal capacity of HEA/IA hydrogels, batch equilibrium tests were conducted. The stock solution of Ni^2+^ ions was prepared by dissolving 1000 mg/L of Ni(NO_3_)_2_·6H_2_O in deionized water.

All batch experiments were conducted by mixing 0.03 g of dried hydrogel with 50 mL of an aqueous solution of Ni(NO_3_)_2_·6H_2_O in 80 mL jars. All solutions were mixed for 48 h at a constant speed of 100 rpm. All experiments were performed in triplicate, and middle values were reported.

The influences of pH (2.0 to 7.0), adsorbent mass (0.008–0.09 g) and ionic strength (0 to 0.3 mol/L) on adsorption were investigated. The pH values of the initial solution were adjusted with dilute NaOH or HNO_3_. For investigating the influence of temperature on nickel adsorption, all experiments were carried out in 10 mg/L Ni^2+^ ion solutions and pH 5.5 at 10, 25 and 50 °C. In order to investigate the influence of contact time on adsorption, adsorption was carried out in time intervals of 0.5–48 h.

In order to investigate the initial concentration of Ni^2+^ ions on adsorption, the concentration ranges from 10 to 500 mg/L were studied.

The adsorption capcity (*q_e_*) was calculated according to the following equation:

(8) $$q_{e}=\frac{\left(C_{o}-C_{e}\right)\cdot V}{m_{s}}$$

where *q_e_* is the adsorption capacity, *C_o_* (mg/L) and *C_e_* (mg/L) are the initial and equilibrium concentration of nickel, *V* (L) is the volume of the solution and *m_s_* (g) is the mass of adsorbent.

### 4.6. Desorption and Regeneration

To determine the efficiency of the desorption process and the possibility of reuse, successive sorption–desorption studies were conducted. By using hydrogel prepared following the synthesis procedure described in this study, three cycles of sorption-desorption experiments were replicated. The desorption of hydrogels was performed with 0.1 M solution of CH_3_COOH, HCl and HNO_3_ (mass of loaded sorbent 0.03 g; the volume of solution 100 mL; duration of process 48 h). In order to examine the influence of pH of the desorption medium on the desorption process, several pH values of the desorption solutions were investigated. In order to examine the potential reusability of hydrogels, three sorption–desorption cycles were repeated on the same adsorbent. A 0.1 M HNO_3_ aqueous solution was used as the desorption medium.

## Figures and Tables

**Table 1:** Parameters of investigated adsorption models for the adsorption of Ni^2+^ ions from aqueous solution onto HEA/IA hydrogels.

MODEL	Equation	Parameter	HEA/10IA
Langmuir	$\frac{1}{q_{e}}=\frac{1}{q_{m}}+\frac{1}{q_{m}K_{L}}\frac{1}{C_{e}}$	*K_L_* (L/g)	5.45
		*q_m_* (mg/g)	269.54
		*R* ^2^	0.9988
		*χ* ^2^	5.0713
Freundlich	$lnq_{e}=lnK_{F}+\frac{1}{n}lnC_{e}$	*K_F_* (mg g^−1^ (L mg^−1^)^1/n^)	2.18
		*N*	1.25
		*R* ^2^	0.9944
		*χ* ^2^	29.5
Redlich–Peterson	$ln\left(K_{R}\frac{C_{e}}{q_{e}}-1\right)=\beta lnC_{e}+lna_{R}$	*K_R_* (L/g)	1.69
		*Β*	0.53
		*a_R_* (Lmol)	0.074
		*R* ^2^	0.9951
		*χ* ^2^	2.2826
Temkin	$q_{e}=\frac{RT}{b}lnK_{t}+\frac{RT}{b}lnC_{e}$	*K_T_* (L/g)	0.10
		*B*	53.92
		*b* (J/mol)	45.95
		*R* ^2^	0.8498
		*χ* ^2^	39.3581
Dubinin–Radushkevich	$lnq_{e}=lnq_{m}-{\beta \varepsilon }^{2}$	*q_m_* (mg g^−1^) × 10^−6^	0.49
		*E* (kJ/mol)	0.64
		*β* × 10^6^	1.23
		*R* ^2^	0.7726
		*χ* ^2^	259.6563

**Table 2:** The *R_L_* values for the HEA/10IA hydrogels at 25 °C.

*C*_0_ (mg/L)	HEA/10IA
10.00	0.9483
53.13	0.7810
98.72	0.6539
202.24	0.4832
307.53	0.3687
427.11	0.2932
518.06	0.2578

**Table 3:** Comparison of maximum adsorption capacities for Ni^2+^ ions on different adsorbents.

Hydrogel	*q_m_* (mg/g)	Reference
HEA/MALA	58.2	[37]
Modified silica	12.4	[38]
C-g-AA	380.1	[39]
Chitosan(chitin)/cellulose composite biosorbents	13.2	[40]
Magnetic p(AMPS) hydrogels	114.9	[41]
HEA/10IA	225.4	Present study

**Table 4:** Kinetic parameters for the adsorption of Ni^2+^ ions onto HEA/10IA hydrogels.

	*q_e_*_,*exp*_ (mg/g)	Pseudo-First Order Model			Pseudo-Second Order Model		
		*k*_1_ (h^−1^)	*q_e__,cal_* (mg/g)	*R* ^2^	*k*_2_ (g mg^−1^ h^−1^)	*q_e__,cal_* (mg/g)	*R* ^2^
HEA/10IA	7.81 ± 0.24	0.123	5.663	0.9299	1.922	8.753	0.9904

**Table 5:** Thermodynamic parameters for adsorption of Ni^2+^ ions.

	Temperature	*q_e,exp_*	∆*G*^o^	∆*H*^o^	∆*S*^o^
	(°C)	(mg/g)	(kJ/mol)	(kJ/mol)	(J/mol/K)
Ni^2+^	10	7.96 ± 0.19	0.208	−4.084	−15.13
	25	7.81 ± 0.24	0.449		
	50	7.44 ± 0.16	0.814		

## Supplementary Materials

The following are available online at https://www.mdpi.com/article/10.3390/gels7040225/s1, Table S1: Experimental plan two factor Optimal (custom) Design of the adsorption capacity of hydrogels in relation to the amount of HEA and IA in the reaction mixture. Table S2: Variance Analysis (ANOVA) for the surface quadratic model of the response to the removal of Ni^2+^ ion from water using the HEA/10IA, Figure S1: RSM surface (a) and contour (b) plot based on the removal of Ni^2+^ ions and analysis of the ANOVA variance of the standard error estimated value (c). Figure S2: RSM surface plot based on the removal of Ni^2+^ ions as a function of mass (g) and pH. Figure S3: RSM surface plot based on the removal of Ni^2+^ ions as a function of pH and t (h). Figure S4: RSM surface plot based on the removal of Ni^2+^ ions as a function of mass (g) and t (h). Figure S5: RSM surface plot based on the removal of Ni^2+^ ions as a function of pH and temperature (°C).
