# Supplementary material for: Removal of Nickel Ions from Aqueous Solutions by 2-Hydroxyethyl Acrylate/Itaconic Acid Hydrogels Optimized with Response Surface Methodology
Source: Gels. 2021 Nov 22;7(4):225. doi: 10.3390/gels7040225 (PMC8628713; doi:10.3390/gels7040225)
Supplement: Supplementary file 1 [file gels-07-00225-s001.zip › gels-1455203-supplementary.pdf]

Supplementary data

Removal of Nickel Ions from Aqueous Solutions by 2-Hydroxyethyl Acrylate/Itaconic Acid Hydrogels optimized with Response Surface Methodology

Katarina Antić [1]*, Antonije Onjia [1], Dana Radović-Vasiljević [2] , Zlate Veličković [3] and Simonida Lj. Tomić [1]

1 Faculty of Technology and Metallurgy, 4 Karnegijeva street, University of Belgrade, Belgrade, Serbia katarina.antic@tmf.bg.ac.rs

2 Faculty of Technology and Metallurgy, 4 Karnegijeva street, University of Belgrade, Belgrade, Serbia onjia@tmf.bg.ac.rs

3 Institute of Chemistry, Technology and metallurgy, 12 Njegoseva Street, University of Belgrade, Belgrade, Serbia dana@nanosys.ihtm.bg.ac.rs

4 University of defense, Military Academy, Veljka Lukica Kurjaka Street 33, 11000 Belgrade, Serbia zlate.velickovic@va.mod.gov.rs

5 Faculty of Technology and Metallurgy, 4 Karnegijeva street, University of Belgrade, Belgrade, Serbia simonida@tmf.bg.ac.rs

2. Results and Discussion

2.1. Optimization of adsorbent preparation

Optimization of the adsorption synthesis was carried out using the surface response methodology (RSM), based on two factors D-optimal design. RSM is in compliance with the principles of environmental protection, where there is a significant decrease in the number of experiments, and consequently a decrease in waste production. The operational values of the selected variables in the experimental plan are given in Table S1, which includes 20 experimental papers plus three replications at the centre point.

Table S1. Experimental plan two factor Optimal (custom) Design of the adsorption capacity of hydrogels in relation to the amount of HEA and IA in the reaction mixture.

Run	m_{HEA} (g)	m_{IA} (g)	Response q_e (mg/g)
1	0.964113	0.0375	5.6
2	0.85	0.01	4.65
3	0.9	0.1	7.81
4	0.98	0.02	5.014
5	0.963956	0.065	5.94
6	0.95887	0.0925	5.91
7	0.8875	0.0925	6.81
8	0.925	0.065	6.79
9	0.85	0.065935	4.9
10	0.926828	0.063663	6.5

11	0.88	0.12	6.12
12	0.925	0.11	6.4
13	0.923725	0.12	6.34
14	0.8875	0.039761	5.46
15	0.917549	0.01	4.9
16	0.85	0.12	4.9
17	1	0.01	4.7
18	0.9	0.1	7.81
19	0.9	0.1	7.81
20	1	0.070464	5.2

Each experiment (except the central point) was performed in duplicate. The output variable was the adsorption capacity. The data obtained in these experiments were fitted with a second-order polynomial equation and the coefficients of the response function and their statistical significance were evaluated by the least square method, using commercial software Design-Expert, Software Version 9 (Stat-Ease, Inc. 2021 E. Hennepin Ave. Suite 480 Minneapolis, USA).

2.3. ANOVA analysis of the variable factors influence on the removal of Ni^{2+} ions from water using HEA/10IA hydrogel

RSM was used to examine the effects of the variables find the optimal combination of variables on the amount of adsorbed metal that will give the highest amount of adsorbed metal. The interactions between process variables and responses were determined by Analysis of variance (ANOVA). Polynomial model terms were evaluated at the 99% confidence level by *P*-value (probability). *P*-Values and q_m (mg/g) value were given in Table S2 for the adsorption of Ni^{2+} ions in the hydrogel. P-Value is the smallest significance level that allows rejection of the null hypothesis. Generally, the smaller the *P*-value is related to the coefficient of the term is more important. When the P-value is <0.05, model and model terms are statistically significant with 95% of the pouch, and when the P-value <0.01 model and model term is significant with 99% reliability [1].

Table S2. Variance Analysis (ANOVA) for the surface quadratic model of the response to the removal of Ni^{2+} ion from water using the HEA/10IA

Terms	P-value	Mean Square	F Value	p-value Prob > F	R-Squared	Adj R-Squared	Pred R-Squared	Adeq Precision
Model	<0.0001	54.25	77.48	< 0.0001	0.9734	0.9675	0.9635	44.695
m	<0.0001	38.43	54.99	< 0.0001				
t	<0.0001	209.75	299.6	< 0.0001				
pH	<0.0001	211.4	301.96	< 0.0001				
T	<0.0605	4.13	5.89	0.179				

The reduced models were obtained by considering the P-value at the 99% confidence level for variables and interactions was statistically significant on metal removal, response surface and contour plots were given to each of Ni^{2+} ions and adsorbent HEA/10IA. The factors showed the effect on q_m (mg/g) according to the second order response function. When the concentration Ni^{2+} ions have a negative effect, the amount of HEA/10IA has a positive effect for adsorbent hg on q_m (mg/g). As shown in calculating qm, since the mass of the adsorbent is inversely proportional to q,

while the initial mass of the adsorbent HEA/10IA, q (mg/g) is reduced. The adsorbent mass coefficient is negative. This is expected, RSM is designed to find the optimal combination of variables that will allow maximum metal removal.

To be significant interaction of factors that can be explained by the quadratic model of the adsorption mechanism for Ni^{2+} ions at the HEA/10IA. The significance of the interaction demonstrated that the adsorption mechanism can be explained by the quadratic model. A model is valid if it can be well predicted. The quality of the fit model, the coefficient of determination (R^2) were evaluated. For an appropriate approach, R squared should be higher than 0.90. R^2 for Ni^{2+} ions is 0.978 respectively, value showed a high correlation between the experimental and the predicted results It illustrates the difference between actual and predicted values. 3-D surface graphs and contour graphics is a graphical representation of the predicted model equations. These graphs were drawn to show the effect of four factors at five levels HEA/10IA mass, pH, time and temperature) on q_m (mg/g).

This analysis provides a better understanding of the impact of factors and their interaction. Each graph shows the effect of both the two factors studied experimental range. Two-dimensional contour plots provide information to estimates that cannot be considered as an experimental combination. The shape of the contour chart shows the property of an interaction. The main and interaction effects of factors are also shown in the response surface plot. The models give the predicted removal efficiency q_m (mg/g) for Ni^{2+} ion as the function of coded variables.

Variation analysis examined the importance of different variables (adsorbent mass, adsorption time, and temperature) on adsorption. Values of "Prob > F" less than 0.0500 indicate model terms are significant. In this case m, t, T are significant model terms. Values greater than 0.1000 indicate the model terms are not significant. The "Pred R-Squared" of 0.9635 is in reasonable agreement with the "Adj R-squared" of 0.9675. "Adeq Precision" measures the signal to noise ratio. A ratio greater than 4 is desirable. A ratio of 44.695 indicates an adequate signal. This model can be used to navigate the design space [2].

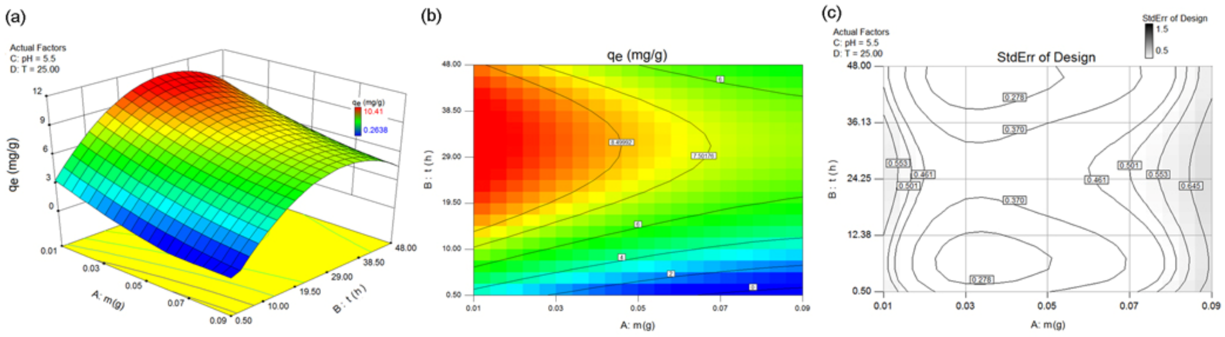

Figure S1. RSM surface (a) and contour (b) plot based on the removal of Ni^{2+} ions and analysis of the ANOVA variance of the standard error estimated value (c).

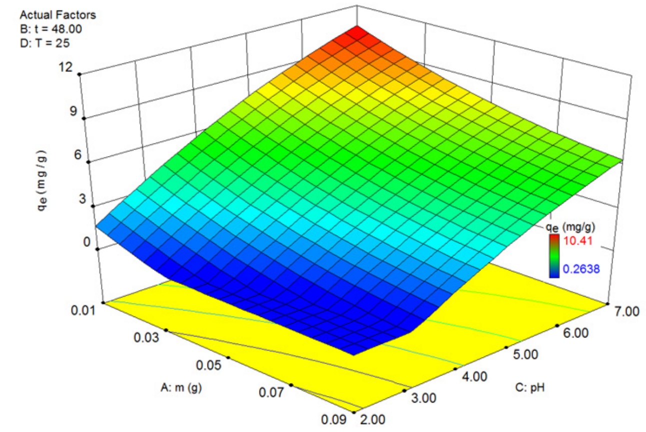

Figure S2. RSM surface plot based on the removal of Ni^{2+} ions as a function of mass (g) and pH

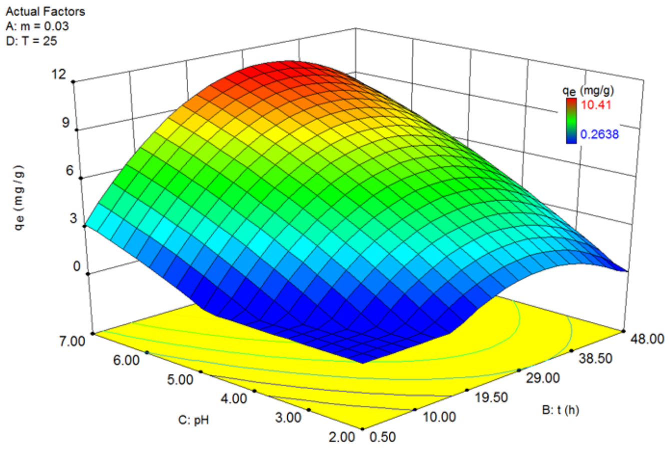

Figure S3. RSM surface plot based on the removal of Ni^{2+} ions as a function of pH and t (h)

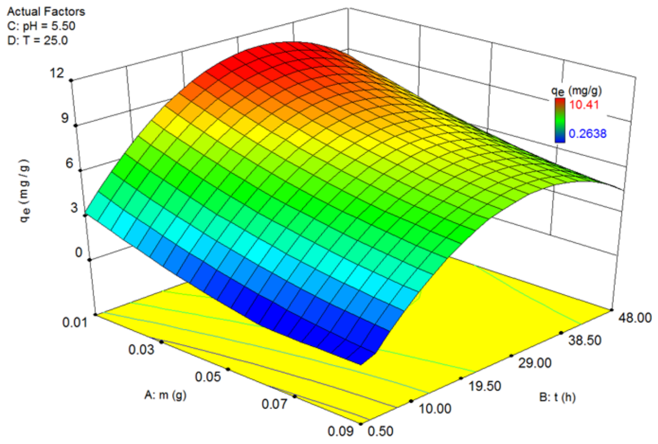

Figure S4. RSM surface plot based on the removal of Ni^{2+} ions as a function of mass (g) and t (h)

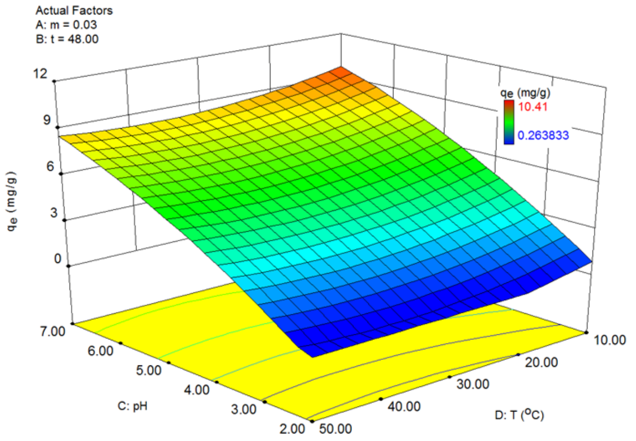

Figure S5. RSM surface plot based on the removal of Ni^{2+} ions as a function of pH and temperature (°C)

1. Bajić, Z.J., Veličković, Z. S., Djokić, V. R., Perić-Grujić, A. A., Ersen, O., Uskoković, P. S., & Marinković, A. D., *Adsorption study of arsenic removal by novel hybrid copper impregnated tufa adsorbents in a batch system.* Clean - Soil, Air, Water, 2016. **44**(11): p. 1477-1488.
2. Xiyili, H., S. Çetintaş, and D. Bingöl, *Removal of some heavy metals onto mechanically activated fly ash: Modeling approach for optimization, isotherms, kinetics and thermodynamics.* Process Safety and Environmental Protection, 2017. **109**: p. 288-300.
